# [Retracted] MicroRNA‑519 enhances HL60 human acute myeloid leukemia cell line proliferation by reducing the expression level of RNA‑binding protein human antigen R

**DOI:** 10.3892/mmr.2024.13335

**Published:** 2024-09-23

**Authors:** Kebin Huang, Bingwei Dong, Yueyue Wang, Tao Tian, Biying Zhang

Mol Med Rep 12: 7830–7836, 2015; DOI: 10.3892/mmr.2015.4455

Following the publication of this paper, it was drawn to the Editor's attention by a concerned reader that certain of the flow cytometric analysis data shown in Fig. 4B on p. 7834 were strikingly similar to data that had already been submitted for publication in different form in another article written by different authors at different research institutes.

Owing to the fact that the contentious data in the above article had already been submitted for publication prior to its submission to *Molecular Medicine Reports*, the Editor has decided that this paper should be retracted from the Journal. The authors were asked for an explanation to account for these concerns, but the Editorial Office did not receive a reply. The Editor apologizes to the readership for any inconvenience caused.

